# Solid-phase extraction and fractionation of multiclass pollutants from wastewater followed by liquid chromatography tandem-mass spectrometry analysis

**DOI:** 10.1007/s00216-022-04066-8

**Published:** 2022-04-23

**Authors:** V. Fernández-Fernández, M. Ramil, R. Cela, I. Rodríguez

**Affiliations:** Department of Analytical Chemistry, Nutrition and Food Sciences, Research Institute On Chemical and Biological Analysis (IAQBUS), Universidade de Santiago de Compostela, 15782 Santiago de Compostela, Spain

**Keywords:** Wastewater, Modular solid-phase extraction, Fractionation, Liquid chromatography tandem-mass spectrometry

## Abstract

Herein, we describe a modular solid-phase extraction (SPE) setup, combining three sorbents, for the effective extraction of neutrals, acidic, and basic micropollutants from wastewater, followed by their further elution in three independent extracts. The performance of this approach was demonstrated for a suite of 64 compounds, corresponding to different chemical families, using liquid chromatography tandem-mass spectrometry (LC–MS/MS). Target compounds were effectively extracted from wastewater samples; moreover, 62 out of 64 species were isolated in just one of the three fractions (neutrals, acids, and bases) obtained from the combination of sorbents. Globally, the efficiency and the selectivity of the SPE methodology improved the features obtained using generic SPE polymers, displaying just reversed-phase interactions. The overall recoveries of the analytical method, calculated against solvent-based calibration standards, stayed between 80 and 120% for 57 and 60 compounds, in raw and treated wastewater, respectively. Procedural limits of quantification (LOQs) varied from 1 to 20 ng L^−1^. Analysis of urban wastewater samples identified a group of 19 pollutants showing either negligible median removal efficiencies (± 20%) during wastewater treatment, or even a noticeable enhancement (case of the biodegradation product of the drug valsartan), which might be useful as markers of wastewater discharges in the aquatic environment.

**Supplementary Information:**

The online version contains supplementary material available at 10.1007/s00216-022-04066-8.

## Introduction

The number of organic compounds of environmental and toxicological concern has increased steadily for the last 20 years. Many of them are introduced in the aquatic environment through urban wastewater [1, 2]. Most of the analytical procedures for the monitoring of these compounds are based on mass spectrometry (MS), combined with different chromatographic techniques, after an extraction and concentration step [3]. In this regard, the hydrophilic-lipophilic balanced (HLB) solid-phase extraction (SPE) sorbents cover the effective extraction of compounds within a broad range of polarities from water samples. Thus, they are usually employed in combination with multianalyte/multiclass liquid chromatography (LC) MS-based methods [4–6]. The price of their high retention efficiency is a limited selectivity, which turns in significant variations in the efficiency of compound ionization (particularly using electrospray ionization, ESI) between sample extracts and solvent-based standards [6]. The so-called matrix effects (MEs) do not only affect the accuracy of the obtained results, but also compound detectability since, in most cases, their ionization efficiency is attenuated for sample extracts when compared to solvent-based standards [5].

Mixed-mode (MM) sorbents, sharing ionic and reversed-phase (RP) interactions, improve the recoveries of highly polar, ionizable compounds in comparison to RP polymers, maintaining an acceptable retention efficiency for neutrals [7, 8]. Moreover, they allow the use of fractionated elution protocols, recovering compounds retained through the RP mechanism and those establishing electrostatic interactions with sorbent in different fractions [9]. Thus, cleaner extracts are obtained for the latter group of compounds, which turns in lower MEs [10–12]. The scientific literature contains previous successful applications of different types of MM sorbents to the selective extraction of basic (i.e., illicit drugs [13] and pharmaceuticals [11]), or acidic compounds (such as anti-hypertension drugs [12] and perfluorinated carboxylic and sulfonic acids [14]) from water samples. Most of these studies have been compiled in a recent review [7]. However, using conventional MM sorbents, the isolation of acidic and basic compounds in two separate fractions, requires two independent SPE extractions, with different MM polymers. In both assays, neutrals are mixed, either with basic or with acidic species.

In order to combine high retention efficiencies during the concentration step with fractionated elution protocols, different alternatives are under evaluation. Zwitterionic MM sorbents permitted the removal of neutrals, in a washing fraction, whilst acids and bases were recovered together [15]. Another possibility involves the combination of different types of sorbents, either packed in the same cartridge, or connected in tandem. This approach was reported to improve the extraction efficiency of low molecular size, polar, and ionizable compounds, poorly retained in RP materials; however, in these previous studies, the sequential elution of compounds in independent fractions was not investigated [8, 16]; consequently, extracts presented a high complexity. Using a multilayered cartridge setup (based on different combinations of conventional MM sorbents), Salas et al. [17] demonstrated the possibility to retain and to recover quantitatively a selection of 13 compounds in neutral, acidic, and basic fractions. In that study, the retention efficiency and the success of the fractionated elution protocol depended on the type of MM sorbents, and their relative proportions in the in-house packed cartridge [17].

The aim of this research was to develop a modular SPE approach, based on tandem combinations of commercially available cartridges, covering the effective extraction and the further fractionated elution of a suite of 64 compounds (log *D* values from − 1.95 to 5.5) attending to their ionizable groups: acids, bases, and neutrals, from wastewater samples. Sorbents were maintained in separate cartridges to increase the versatility of the elution protocol. Each SPE fraction was analyzed using different LC–MS/MS procedures, finely tuned to enhance compound detectability and to reduce blank contamination problems. The performance of the method was characterized in terms of extraction efficiency, MEs, and accuracy. Thereafter, it was applied to determine the levels of target compounds in raw and treated wastewater samples obtained from urban sewage treatment plants (STPs).

## Material and methods

### Solvents, sorbents, and standards

Methanol (MeOH) and acetonitrile (ACN), both LC–MS grade; formic acid (FA, LC–MS grade); ammonia (NH_3_, 7 M solution in MeOH); and ammonium fluoride (NH_4_F) were purchased from Merck (Darmstadt, Germany). Ultra-pure deionized water (18.2 MΩ cm^−1^) was obtained from a Genie U system (Rephile, Shanghai, China).

RP OASIS HLB 60-mg and 200-mg cartridges, and 150-mg MM cartridges (MCX, RP, and strong cationic exchange sorbent; and WAX, RP, and weak anionic exchange sorbent) were provided by Waters (Milford, MA, USA). Ionic exchange 500-mg cartridges containing either sulfonic functionalities (SCX), or quaternary amines (SAX), as charged groups bonded to silica particles, were obtained from Agilent (Santa Clara, CA, USA).

Native standards of species involved in this research were purchased from Sigma-Aldrich (St. Louis, MO, USA). Compounds were selected attending to their environmental and/or toxicological concern, including species compiled in the 2020 revision of the EU Watch List of contaminants to control in the aquatic environment [18]. The suite of compounds includes species without ionizable groups (neutrals, i.e., organophosphorus compounds and certain neonicotinoids), weak (phenols) and strong acids (carboxylic, tetrazolic, sulfonic, etc.), weak and strong bases (i.e., azoles and tertiary amines, respectively), and pollutants combining acidic and basic functionalities in the same molecule (e.g., certain angiotensin receptor antagonists, ARA-II, as losartan). The list of analytes, including their log *D* values and their categorization as acids, bases, or neutrals, is given in Table 1. Individual stock solutions of each compound were prepared in MeOH, except in case of neonicotinoids (ACN). Further dilutions and mixtures were also made in MeOH. Stock solutions and diluted mixtures were maintained at − 20 °C and used throughout this study. The exception was perfluorinated carboxylic acids. Their methanolic solutions were renewed every month to prevent esterification of the carboxylic moiety [19].

**Table 1:** Summary of target compounds, including LC–ESI–MS/MS determination conditions, instrumental LOQs, and linearity evaluation

**Group**	**Compound name**	**Precursor ion**	**Q1 (CE)**	**Q2 (CE)**	**Ratio (Q2/Q1)**	**Ret time (min)**	**IS**	**ESI**	Log *D* (pH 7)	LOQs (ng/mL)^a^	Linearity (*R*^2^, 0.5–300 ng mL^−1^)
Acids	2,4-Dichlorophenoxyacetic acid	219.0	161.0 (12)	125.0 (32)	0.08	6.66	2,4-Dichlorophenoxyacetic acid-d_5_	-	− 1.14	2	0.994^b^
	4-(2,4-Dichlorophenoxy)butyric acid	247.0	161.0 (4)	35.0 (52)	0.11	9.54	2,4-Dichlorophenoxyacetic acid-d_5_	-	0.97	2	0.990^b^
	Candesartan	441.1	235.1 (20)	192.1 (32)	0.90	8.17	Irbesartan-d_4_	+	0.59	0.3	0.993
	Eprosartan	425.1	135.1 (36)	97.1 (28)	0.30	8.10	Irbesartan-d_4_	+	− 0.57	0.3	0.997
	Fenoprop	267.0	195.0 (12)	159.0 (32)	0.20	8.57	Irbesartan-d_4_	-	− 0.13	0.5	0.993
	Irbesartan	429.3	207.1 (24)	195.2 (24)	0.17	9.71	Irbesartan-d_4_	+	3.31	0.1	0.998
	Losartan	423.2	207.1 (28)	405.2 (8)	0.37	9.22	Irbesartan-d_4_	+	1.51	0.2	0.997
	2-Methyl-4-chlorophenoxyacetic acid	199.0	140.8 (12)	35.1 (48)	0.15	6.85	2,4-Dichlorophenoxyacetic acid-d_5_	-	− 1.3	1	0.996
	Mecoprop	213.0	140.8 (12)	35.1 (48)	0.13	7.68	2,4-Dichlorophenoxyacetic acid-d_5_	-	− 0.92	1	0.996
	Olmesartan	447.2	207.1 (24)	429.2 (8)	0.34	7.60	Irbesartan-d_4_	+	− 0.78	0.2	0.997
	Pentafluoropropanoic acid	163.0	118.8 (8)	68.9 (40)	0.02	1.18	Perfluorooctanoic acid ^13^C_2_	-	− 1.33	0.2	0.999
	Perfluorobutanoic acid	213.0	169.0 (5)	n.a	-	2.27	2,4-Dichlorophenoxyacetic acid-d_5_	-	− 0.36	0.1	0.999
	Perfluorobutano sulfonic acid	299.0	80.0 (41)	99.0 (33)	0.37	6.99	2,4-Dichlorophenoxyacetic acid-d_5_	-	− 1.81	0.1	0.997
	Perfluorooctanoic acid	413.0	369.0 (17)	169.0 (5)	0.29	9.90	Perfluorooctanoic acid ^13^C_2_		2.69	0.1	0.996
	Perfluorooctano sulfonic acid	499.0	80.0 (49)	99.0 (60)	0.20	10.45	Perfluorooctano sulfonic acid ^13^C_8_	-	1.01	0.4	0.998
	Telmisartan	515.1	497.2 (40)	276.1 (40)	0.45	9.72	Irbesartan-d_4_	+	3.65	0.2	0.996
	Valsartan	436.2	207.1 (32)	235.1 (20)	0.95	9.00	Irbesartan-d_4_	+	− 0.68	0.2	0.996
	Valsartan acid	267.1	206.1 (20)	178.1 (36)	0.55	6.62	Valsartan acid-d_4_	+	− 1.95	0.1	0.998
Bases	Acetamiprid^c^	223.1	126.0 (27)	56.1 (12)	0.41	5.69	Acetamiprid-d_3_	+	1.55	0.1	0.999
	Amitriptyline	278.2	233.1 (16)	91.1 (36)	1.04	7.76	Imazalil-d_5_	+	2.28	0.1	0.998
	Citalopram	325.2	109.1 (28)	262.1 (16)	0.28	6.53	Flecainide-d_4_	+	1.02	0.1	0.997
	Climbazole	293.1	197.1 (16)	141.0 (24)	0.21	7.54	Climbazole-d_4_	+	3.47	0.1	0.999
	Clomipramine	315.2	86.1 (20)	58.1 (56)	0.75	8.34	Clotrimazole-d_5_	+	2.6	0.1	0.999
	Cloperastine	330.2	201.1 (16)	166.1 (40)	0.62	8.01	Clotrimazole-d_5_	+	2.9	0.1	0.998
	Clotrimazole	277.1	239.1 (60)	165.0 (28)	0.42	8.07	Clotrimazole-d_5_	+	4.87	0.1	0.998
	Fenticonazole	454.9/456.9	198.9 (36)	198.9 (36)	0.63	10.10	Miconazole-d_5_	+	4.56	0.1	0.998
	Flecainide	415.1	398.1 (24)	301.0 (40)	0.59	6.63	Flecainide-d_4_	+	0.72	0.1	0.999
	Fluconazole	307.1	219.9 (20)	70.0 (44)	0.55	5.23	Tramadol ^13^C d_3_	+	0.45	0.2	0.998
	Haloperidol	376.2	123.0 (44)	165.1 (24)	0.96	7.03	Flecainide-d_4_	+	2.58	0.1	0.999
	Imazalil	297.1	255.0 (12)	158.9 (20)	4.00	7.38	Imazalil-d_5_	+	3.37	0.5	0.996
	Imidacloprid^c^	256.1	175.1 (12)	209.0 (12)	1.00	4.62	Imidacloprid-d_4_	+	0.07	0.4	0.999
	Lamotrigine	256.0	43.1 (40)	210.8 (32)	0.20	4.88	Lamotrigine ^13^C_3_	+	1.23	0.5	0.999
	Metconazole	320.1	70.0 (28)	124.9 (52)	0.09	10.31	Tebuconazole-d_9_	+	3.72	0.3	0.999
	Miconazole	417.0	158.8 (40)	160.8 (36)	0.94	9.51	Miconazole-d_5_	+	4.81	0.2	0.999
	Myclobutanil	289.1	70.1 (16)	125.1 (32)	0.26	9.27	Myclobutanil-d_4_	+	3.07	0.1	0.999
	N-Desethyl amiodarone	617.9	72.1 (28)	546.9 (24)	0.47	10.15	Miconazole-d_5_	+	5.51	0.1	0.999
	N-Desmethyl citalopram	311.2	108.9 (28)	262.1 (16)	0.41	6.58	Venlafaxine-d_6_	+	1.02	0.2	0.998
	Norsertraline	275.0	158.8 (20)	129.1 (30)	0.10	8.38	Norsertraline ^13^C_6_	+	2.8	0.5	0.999
	O-Desmethyl venlafaxine	264.2	58.1 (17)	246.2 (13)	0.25	4.59	Venlafaxine-d_6_	+	− 0.37	0.1	0.997
	Penconazole	284.1	70.1 (15)	159.0 (30)	0.47	9.98	Tebuconazole-d_9_	+	4.64	0.1	0.998
	Prochloraz	376.0	308.0 (4)	70.1 (24)	0.82	9.78	Myclobutanil-d_4_	+	4.59	0.2	0.998
	Propiconazole	342.1	159.0 (32)	69.1 (16)	0.79	10.17	Myclobutanil-d_4_	+	3.65	0.2	0.999
	Propranolol	260.2	116.1 (20)	183.1 (20)	0.50	6.33	Tramadol ^13^C-d_3_	+	0.45	0.2	0.998
	Sertaconazole	437.0/439.0	180.9 (40)	180.9 (36)	0.64	9.50	Miconazole-d_5_	+	5.6	0.1	0.999
	Sertraline	306.1	158.9 (36)	275.0 (12)	0.70	8.25	Norsertraline ^13^C_6_	+	2.7	0.2	0.999
	Tebuconazole	308.1	70.0 (40)	124.9 (47)	0.09	10.07	Tebuconazole-d_9_	+	3.77	0.2	0.999
	Terbutryn	242.1	185.9 (20)	68.0 (60)	0.35	8.07	Imazalil-d_5_	+	3.38	0.1	0.999
	Tetraconazole	372.0	158.9 (32)	70.0 (24)	0.93	9.59	Myclobutanil-d_4_	+	3.56	0.2	0.997
	Thiabendazole	202.0	175.0 (28)	131.1 (40)	0.75	3.97	Tramadol ^13^C d_3_	+	2.47	0.1	0.998
	Tioconazole	386.9	130.9 (32)	68.9 (24)	0.06	8.76	Miconazole d_5_	+	4.11	0.5	0.999
	Tramadol	264.2	58.1 (20)	n.a	-	4.79	Tramadol ^13^C d_3_	+	− 0.06	0.1	0.996
	Trazodone	372.2	176.1 (24)	147.9 (40)	0.77	5.73	Tramadol ^13^C d_3_	+	2.41	0.3	0.997
	Venlafaxine	278.2	58.1 (25)	260.2 (9)	0.25	6.08	Venlafaxine-d_6_	+	0.39	0.1	0.999
Neutrals	Clothianidin	250.0	169.1 (8)	131.9 (8)	0.60	5.32	Chlothianidin-d_3_	+	− 1.26	0.2	0.999
	Cresyl diphenyl phosphate	341.1	90.9 (44)	151.9 (48)	0.60	9.20	Tributyl phosphate-d_27_	+	4.76	0.3	0.999
	Dimoxystrobin	327.2	205.1 (8)	116.05 (24)	1.05	8.47	Tributyl phosphate-d_27_	+	5.09	0.1	0.998
	Octyl isothiazolinone	214.1	101.9 (16)	43.1 (28)	0.50	7.91	Triclosan ^13^C_6_	+	3.69	0.1	0.995
	Tris(2-Chloroethyl) phosphate	284.9	98.9 (20)	124.9 (16)	0.85	5.81	Tris(1-Chloro-2-propyl) phosphate-d_18_	+	1.47	1	0.998
	Tris(1-Chloro-2-propyl) phosphate	327.0	98.9 (28)	174.9 (12)	0.33	7.85	Tris(1-Chloro-2-propyl) phosphate-d_18_	+	2.53	0.1	0.992
	Triclosan	286.8/288.9	35.1 (5)	35.1 (5)	0.65	9.05	Triclosan ^13^C_6_	-	5.28	0.3	0.999
	Thiamethoxam	292.0	211.1 (8)	132.0 (24)	0.40	5.00	Thiamethoxam-d_4_	+	0.16	0.1	0.999
	Tributoxyethyl phosphate	399.3	299.2 (13)	199.1 (13)	0.97	9.33	Tributyl phosphate-d_27_	+	3.28	0.1	0.994
	Tributyl phosphate	267.1	98.8 (20)	80.9 (60)	0.22	8.91	Tributyl phosphate-d_27_	+	3.83	0.1	0.992
	Triphenyl phosphate	327.1	77.0 (28)	152.1 (48)	0.40	8.86	Tributyl phosphate-d_27_	+	4.59	0.2	0.993

^a^Instrumental LOQs

^b^*R*^2^ values for standards in the range of concentrations from 2 to 300 ng mL^−1^

^c^Transitions of these compounds were included also in the group of neutrals

A selection of isotopically labeled compounds (either deuterated or ^13^C species) was obtained from Merck and Toronto Research Chemicals (North York, Canada), either as pure compounds, or as stocks in MeOH (usually 100–1000 μg mL^−1^, Table S1). Mixtures of these species were also made in MeOH. They were added to water samples, as surrogate standards (SSs), before SPE extraction.

Solvent-based calibration standards were prepared in MeOH, or MeOH to FA (99:1) case of acidic species, in the range of concentrations from 0.5 to 300 ng mL^−1^. The concentration of SSs in calibration standards was 50 ng mL^−1^.

### Samples and sample preparation

Wastewater was obtained from four different urban STPs in Galicia (Northwest Spain). All of them apply similar wastewater treatments involving primary and biological (activated sludge) units. Grab samples were used during method development. Integrated (24-h time proportional) samples were employed to measure the concentrations of target compounds, and to evaluate their removal efficiencies during wastewater treatment. Samples were received in glass bottles, sequentially passed through quartz (0.7-μm cutoff) and cellulose acetate filters (0.45-μm pore-size), and stored at 4 °C, for a maximum of 24 h, before extraction.

**Fig. 1:**
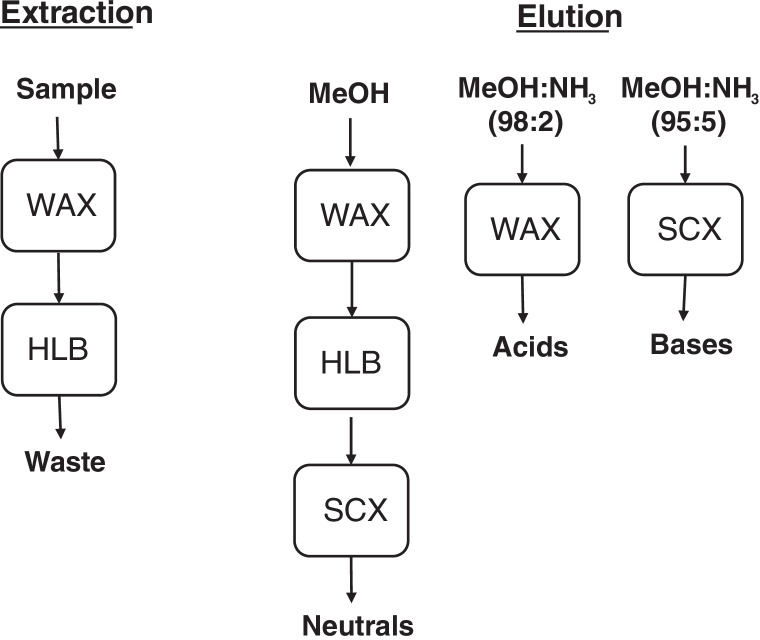
Scheme of sample concentration and elution steps in the modular solid-phase extraction procedure

During method development, different combinations of sorbents were tested. Under final working conditions, a modular SPE setup consisting of a MM 150 mg WAX cartridge (top) on-line connected to a RP 60 mg HLB one (bottom) was employed. Samples (100 mL volume aliquots), spiked with SSs and adjusted at neutral pH (6.5–7.5) when required, were passed through both cartridges at a flowrate of c.a. 5 mL min^−1^. After washing sample containers and connections with SPE sorbents, using 10 mL of ultrapure water, cartridges were dried using a gentle stream of nitrogen and connected to an ionic exchange (SCX) one, previously conditioned with MeOH. Neutrals and weak acids were recovered with MeOH flowing through the three sorbents (extract volume 5 mL). After disconnecting the three cartridges, compounds with a strong acidic functionality (carboxylic, sulfonic, or tetrazolic groups) were recovered from the WAX cartridge with 2 mL of MeOH to NH_3_ (98:2). Basic species were eluted from the SCX one using 5 mL of MeOH to NH_3_ (95:5) (Fig. 1). Every extract was evaporated and adjusted to a final volume of 1 mL; moreover, that from the WAX sorbent was acidified with 0.020 mL of FA. Reference SPE extractions were carried out using RP HLB cartridges (200 mg sorbent), for the concentration of 100 mL samples. In this case, all compounds were recovered in the same fraction of methanol (5 mL), which was further concentrated to 1 mL. Extracts were filtered (0.22-μm pore-size syringe filter) before LC–MS/MS analysis.

### LC–MS/MS determination conditions

Compounds were determined using an ultra-performance liquid chromatography (UPLC) triple quadrupole-type MS system provided by Agilent. The UPLC was 1290 Infinity II connected through a jet-stream ESI source to an i-funnel Agilent 6495 QqQ instrument. Different analytical (LC or UPLC) and delay columns were employed for the separation of target compounds, and to discriminate responses for contaminants existing in the mobile phase from those corresponding to injected compounds. Detailed UPLC conditions for each group of compounds, including type of columns, mobile phase composition, flowrate, and column temperature are compiled in Table S2. The injection volume was set at 2 μL in all methods. Voltages of the ESI source were 3000 V and 2000 V for positive and negative ionization modes, respectively. The fragmentor voltage was 166 V and the MRM parameters for each compound, including ionization mode and ratio between qualification (Q2) and quantification (Q1) transitions, are compiled in Table 1. In a few cases (e.g., perfluorobutanoic acid and tramadol, TRA), only one transition was available. MRM parameters for compounds employed as SSs are given in Table S1.

In addition to the QqQ system, a time-of-flight (TOF) instrument (Agilent 6550) was employed to investigate the distribution of additional compounds in the SPE fractions obtained from non-spiked wastewater samples. In this case, the pseudo-molecular ions ([M + H]^+^ or [M − H]^−^) of each species were extracted using a mass window of 20 ppm. Compound identities were further confirmed against authentic standards. In this case, the employed LC conditions were those reported in Table S2 for basic species.

### Extraction efficiency, matrix effects, and accuracy evaluation

The extraction efficiency (EEs, %) of the modular SPE protocol described in “Samples and sample preparation” (accounting for yields of extraction, fractionated elution, and extract concentration to 1 mL) was assessed as the ratio of responses (peak area for the Q1 transition without SSs correction) obtained for spiked wastewater aliquots and spiked SPE extracts multiplied by 100. Matrix effects (MEs, %) during ESI were evaluated comparing the difference of responses for spiked and non-spiked extracts of each sample (raw and treated wastewater) with those observed for a solvent-based standard of the same concentration. Values close to 100% correspond to similar ionization efficiencies for sample extracts versus solvent-based standards. On the other hand, normalized response ratios below and above 100% mean suppression and enhancement of compound ionization in sample extracts versus solvent-based standards [20]. The above parameters (EEs and MEs) were evaluated using an additional level of 1 ng mL^−1^ referred to the water sample (equivalent to 100 ng mL^−1^ in the corresponding SPE extract).

The accuracy of the final procedure was investigated using samples spiked at three levels: 50, 200, and 1000 ng L^−1^. For each type of wastewater, non-spiked (*n* = 3 replicates) and spiked fractions (*n* = 3, for each addition level) were fortified with SSs (500 ng L^−1^) and processed as reported in “Samples and sample preparation.” Responses obtained for each compound were corrected with that measured to the assigned SS (Table 1), and compared to those obtained for solvent-based standards (concentration range from 0.5 to 300 ng mL^−1^).

Two different kinds of blanks were considered during method development and application. Instrumental blanks corresponded to simulated (false) injections. That is, the injection valve changes from the by-pass to the main-pass position, with the mobile phase flowing through the injector loop and the injection needle to the LC column; however, the autosampler does not select any vial (sample, procedural blank, standard or solvent). These experiments permitted identifying contamination problems related to the UPLC system and/or the mobile phase (mainly the aqueous phase). Procedural blanks were prepared using ultrapure water samples, spiked only with the selection of SSs, and submitted to the adopted modular SPE protocol. This type of blanks is useful to detect contamination problems related to the sample preparation process.

Instrumental limits of quantification (LOQs) were calculated as the concentration of each compound producing a response with a signal to noise ratio (*S*/*N*) of 10 for the less intense of the selected transitions (usually Q2) in solvent-based standards. Procedural LOQs were estimated from instrumental LOQs, considering a 100-fold concentration factor, corrected with EEs and MEs when they were outside the range of values between 80 and 120%. Moreover, for compounds found in the procedural blanks, the LOQs of the method were calculated as the average concentration measured in blank extracts plus10 times its standard deviation.

## Results and discussion

### LC–ESI–MS/MS conditions

Three LC-QqQ-MS methods were employed to enhance the detectability of each group of considered compounds (acids, bases, and neutrals). In case of IMI and ACE, their transitions were included in methods developed for neutral and basic species. Except for TCS, the rest of neutrals and bases were determined in ESI ( +); thus, FA was used as mobile phase modifier (0.1%) to promote their ionization. ACN, instead of MeOH, was preferred as organic mobile phase to reduce the retention of some highly lipophilic organophosphate flame retardants included in the group of neutrals, and to decrease the pressure in the UPLC system considering that two identical columns (delay and analytical columns) were required to cope with instrumental blanks noticed for some compounds within this group.

As regards acidic compounds, FA (0.1%) and NH_4_F (1 mM) were tested as mobile phase additives. The ARA-II drugs showed a higher ionization efficiency under ESI ( +). On the other hand, herbicides and perfluorinated compounds led only to their [M − H]^−^ ions. Thus, ESI ( +) and ESI ( −) modes were combined in this method. Depending on the type of modifier, differences between 20 and 40% in responses obtained for herbicides and perfluorinated compounds were noticed. However, most ARA-II drugs rendered one order of magnitude higher responses using NH_4_F as modifier (Fig. S1).

The instrumental LOQs of compounds considered in this research were not only conditioned by their ionization efficiencies, but also by the existence of instrumental contamination sources. These problems were noticed in LC–MS/MS records obtained for simulated injections. Particularly, mobile phases contributed significantly to the presence of several perfluorinated and organophosphate compounds in instrumental blanks. To discriminate the response due to instrumental contamination from that of the standard, delay columns were connected between the mobile phase mixer and the injector of the UPLC system. These columns are described in Table S2. Fig. S2 illustrates the separation of the chromatographic peak (earlier signal) for a low concentration standard of selected compounds: tris(2-chloro-isopropyl) phosphate (TCPP), tributoxyethyl phosphate (TBEP), and perfluorooctanoic acid (PFOA), from that corresponding to mobile phase contamination (latter peak), after installing the corresponding delay column.

Under final working conditions, the LC–ESI–MS/MS methods achieved instrumental LOQs in the range from 0.1 to 0.5 ng mL^−1^ for most of the target compounds. In all cases, linear responses were attained for concentrations up to 300 ng mL^−1^ (Table 1).

### Solid-phase extraction and fractionated compound elution

Preliminary SPE experiments were carried out using spiked aliquots of ultrapure water, considering different combinations of sorbents. The first tested setup involved retention of the suite of compounds (neutrals, acids, and bases) in a MM MCX sorbent [9]. In this case, samples were adjusted at pH 3 to improve the retention of highly polar, acidic species, in the MCX sorbent, through RP interactions. Neutrals are expected to be retained by the same mechanism and bases through electrostatic interactions with negatively charged sites of the polymer. Obviously, the latter interactions are favored in acidified samples. During the elution step, the MCX cartridge was connected to an anionic exchange (SAX) one. Distribution of compounds was investigated in the following solvent fractions: MeOH (5 mL) flowing through both cartridges connected in series, MeOH to NH_3_ (95:5) recovered from the upper MCX sorbent (after removing the SAX one), and MeOH to FA (95:5) collected from the SAX cartridge. Under these conditions, neither the retention nor the fractionation of neutrals and acidic compounds was satisfactory. As example, the short-chain perfluorinated compounds (C_3_ carboxylic acid and C_4_ carboxylic and sulfonic acids) were not retained by the MCX sorbent; so, their EEs remained below 20%. Compounds with acidic and basic moieties in their structures (i.e., most of the ARA-II pharmaceuticals) were found in the fraction of basic drugs, whilst acidic drugs (e.g., valsartan, VAL, and valsartan acid, VALA), herbicides (phenoxy acids), and C_8_ perfluorinated compounds eluted together with neutrals in the methanolic fraction. That is, they were not fractionated from neutrals by the SAX sorbent. To sum up, this setup did not show any advantage compared to the single use of the MM MCX sorbent, enabling the fractionated elution of bases from neutral and acids.

The second SPE setup considered concentration of water samples, at neutral pH, using a weak anionic exchange MM sorbent (WAX) [12]. In the elution step, this sorbent was connected to a pure cationic exchanger (SCX) cartridge. As in the former case, three different fractions were collected. MeOH was passed through both cartridges connected in series to recover neutrals. Thereafter, they were disconnected and eluted with MeOH to NH_3_ (98:2). Above 95% of the responses (peak areas) observed for the suite of selected compounds was noticed in the expected SPE fraction accordingly to their preliminary classification given in Table 1. The only exceptions were the neonicotinoid insecticides imidacloprid (IMI) and acetamiprid (ACE), distributed between the neutral and the basic fractions in similar percentages.

On view of these preliminary results, the second setup was adopted, and retention and elution conditions were re-evaluated using spiked wastewater samples. Some highly polar and basic species, such as TRA, venlafaxine (VEN) and O-desmethyl venlafaxine (O-DVEN), citalopram (CIT), and N-desmethyl citalopram (N-DCIT) (their log *D* values ranged from − 0.4 to 1.0 at neutral pH, Table 1), were not retained quantitatively by the WAX sorbent. For 100 mL volume wastewater samples, between 5 and 18% of the responses measured for these compounds were noticed in the extract from a second WAX cartridge on-line connected to the first one. In order to improve their retention, the mixed-mode WAX cartridge was combined (placed on top) with 60 mg HLB one to reinforce the RP retention mechanism during sample concentration. As regards the volume and the type of solvents employed in the fractionated elution protocol, 5 mL of MeOH was passed through the ternary combination of sorbents (MM, RP, and strong cationic exchange) to recover neutrals (Fig. 1). Triclosan (TCS), selected as representative of weak acidic phenolic species (predicted pKa 7.8), was also quantitatively eluted in this fraction. Again, IMI and ACE were partially retained by the SCX sorbent, being detected in neutral and, mostly, basic fractions. The other two neonicotinoids included in the study (thiamethoxam, THM, and clothianidin, CLO) were found only in the neutral fraction (methanolic extract). Likely, the chloronicotinic ring existing in the structures of IMI and ACE leads to a weak interaction of both compounds with the strong anionic exchange sorbent. None of the tested acidic compounds was released from the WAX cartridge during elution of neutrals. So, the HLB cartridge was discarded after this step (Fig. 1). Acids were recovered using just 2 mL of MeOH with a 2% of NH_3_, which is in agreement with the data published by G. Castro and co-workers [12] for SPE of ARA-II species using WAX cartridges. Finally, basic compounds showed a strong interaction with the SCX sorbent. Their quantitative elution (particularly in case of those containing tertiary amine groups) was required to increase the percentage of NH_3_ added to MeOH from 2 to 5%, using 5 mL of this mixture.

### Performance of the method

The EEs of the sample preparation process, calculated as defined in “Extraction efficiency, matrix effects, and accuracy evaluation,” are summarized in Table 2. For most compounds, EEs ranged from 80 to 120%. In a few cases, values between 70 and 130% were noticed. On the other hand, six compounds showed non-quantitative extraction yields. Within the group of bases, EEs around 50% were observed for the pharmaceuticals: fenticonazole, miconazole, and sertaconazole, and the drug metabolite N-desethyl amiodarone. The four are relatively lipophilic compounds, with log *D* values above 4.5 (Table 1). Very likely, non-quantitative EEs are the result of sorption losses on glassware and connections with SPE cartridges. Although it was attempted to improve their recoveries by addition of MeOH to the water samples (10–20 mL of methanol per 100 mL of sample), this approach led to retention problems for polar basic species, positively charged at neutral pH values. Since the latter ones have a higher potential to be present in the water phase than more lipophilic drugs, no organic solvent was added to samples before SPE. The 2nd group of compounds displaying non-satisfactory recoveries was the neonicotinoids IMI and ACE. As commented in “LC–ESI–MS/MS conditions,” both species were distributed between neutral and basic fractions. In raw wastewater, the overall EEs for each group of pollutants were 94% (acids), 91% (bases) and 86% (neutrals). For treated wastewater, average SPE EEs were 96%, 76%, and 94% for acids, bases, and neutrals, respectively.

**Table 2:** Extraction efficiencies for spiked samples of raw and treated wastewater using the modular SPE procedure, *n* = 3 replicates

Group	Compound	Raw wastewater		Treated wastewater		Group	Compound	Raw wastewater		Treated wastewater	
		Mean	RSDs (%)	Mean	RSDs (%)			Mean	RSDs (%)	Mean	RSDs (%)
Acids	2,4-Dichlorophenoxyacetic acid	97%	3%	102%	1%	Bases	Metconazole	97%	4%	84%	4%
	4-(2,4-Dichlorophenoxy) butyric acid	97%	7%	99%	5%		Miconazole	52%	19%	67%	1%
	Candesartan	88%	2%	99%	2%		Myclobutanil	102%	4%	88%	5%
	Eprosartan	97%	3%	96%	2%		N-Desethyl amiodarone	42%	22%	64%	4%
	Fenoprop	101%	5%	100%	2%		N-Desmethyl citalopram	93%	3%	81%	5%
	Irbesartan	92%	2%	97%	2%		Norsertraline	75%	8%	75%	4%
	Losartan	88%	3%	95%	4%		O-Desmethyl venlafaxine	118%	3%	94%	4%
	2-Methyl-4-chlorophenoxyacetic acid	98%	6%	97%	3%		Penconazole	103%	3%	85%	5%
	Mecoprop	98%	8%	101%	3%		Prochloraz	90%	5%	79%	4%
	Olmesartan	98%	3%	99%	2%		Propiconazole	97%	4%	85%	5%
	Pentafluoropropanoic acid	83%	3%	82%	4%		Propranolol	101%	3%	82%	4%
	Perfluorobutanoic acid	88%	3%	100%	2%		Sertaconazole	42%	29%	65%	2%
	Perfluorobutano sulfonic acid	92%	4%	102%	3%		Sertraline	90%	3%	77%	2%
	Perfluorooctanoic acid	86%	1%	116%	5%		Tebuconazole	98%	2%	84%	4%
	Perfluorooctano sulfonic acid	88%	4%	75%	2%		Terbutryn	100%	3%	79%	3%
	Telmisartan	92%	3%	97%	2%		Tetraconazole	100%	4%	86%	5%
	Valsartan acid	104%	3%	84%	4%		Thiabendazole	128%	15%	85%	5%
	Valsartan	106%	2%	94%	3%		Tioconazole	71%	9%	70%	2%
Bases	Acetamiprid	61%	19%	57%	17%		Tramadol	113%	3%	87%	5%
	Amitriptyline	102%	4%	79%	3%		Trazodone	94%	4%	75%	5%
	Citalopram	105%	5%	82%	4%		Venlafaxine	112%	3%	86%	5%
	Climbazole	96%	3%	82%	4%	Neutrals	Clothianidin	94%	4%	95%	2%
	Clomipramine	94%	3%	73%	3%		Cresyl diphenyl phosphate	80%	8%	84%	5%
	Cloperastine	91%	4%	20%	31%		Dimoxystrobin	89%	5%	99%	4%
	Clotrimazole	80%	6%	71%	2%		Octyl isothiazolinone	81%	7%	85%	7%
	Fenticonazole	57%	26%	53%	5%		Tris(2-Chloroethyl) phosphate	87%	7%	101%	2%
	Flecainide	110%	5%	87%	4%		Tris(1-Chloro-2-propyl) phosphate	85%	13%	106%	4%
	Fluconazole	105%	4%	87%	5%		Triclosan	87%	12%	79%	5%
	Haloperidol	109%	7%	79%	4%		Thiamethoxam	86%	3%	96%	2%
	Imazalil	102%	6%	80%	3%		Tributoxyethyl phosphate	93%	7%	105%	5%
	Imidacloprid	45%	15%	56%	21%		Tributyl phosphate	77%	10%	96%	3%
	Lamotrigine	118%	5%	88%	4%		Triphenyl phosphate	90%	19%	92%	4%

**Fig. 2:**
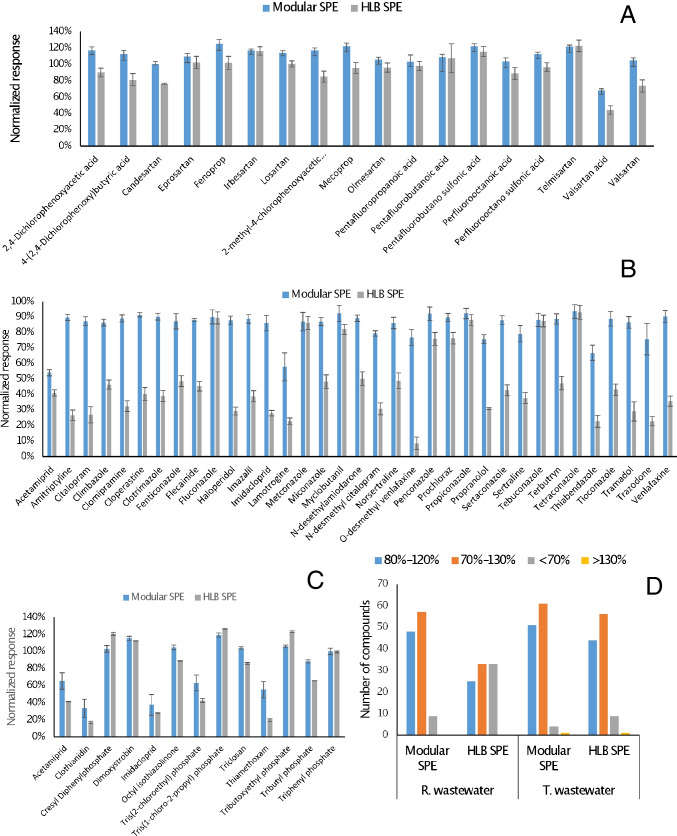
Comparison of matrix effects (MEs, %) obtained for the different groups of compounds using modular and direct (reversed-phase extraction) SPE approaches for raw wastewater concentrated 100 times. A Acids. B Bases. C Neutrals. D Distribution of ME values in raw and treated wastewater as function of the solid-phase extraction protocol

The selectivity of the modular SPE methodology was assessed comparing the responses obtained for the three groups of compounds in spiked extracts from raw and treated wastewater, with those corresponding to solvent-based standards prepared in MeOH [20]. Moreover, the normalized response ratios were compared to those obtained using a HLB sorbent, applied to 100 mL aliquots of the same water samples. In case of acids, most species showed normalized responses in the range from 80 to 120% (Fig. 2A). The only exception was VALA affected by moderate (68%) and strong signal suppression (44%) effects in the modular SPE and HLB extracts, respectively (Fig. 2A). It is worth noting that, for acidic compounds, the RP methodology (based on the use of an HLB 200-mg cartridge for concentration of 100-mL samples) failed to recover the short-chain perfluorinated compounds (perfluoropropanoic, perfluorobutanoic, and perfluorobutane sulfonic acids), data not shown. For the set of basic species (including the neonicotinoids ACE and IMI), significantly higher signal suppression effects were noticed for most compounds using the non-selective HLB extraction protocol, than following the modular approach (Fig. 2B). Finally, for neutrals, only CLO and THM presented strong signal suppression effects (normalized responses below 60%, Fig. 2C). The magnitude of this attenuation was significantly higher for HLB extracts than for those obtained with the combination of sorbents described in this research. In summary, for the raw wastewater matrix, 48 out of 64 compounds showed low MEs (normalized responses from 80 to 120%) using the modular SPE procedure, whilst only 25 species were within this interval with the SPE sorbent (Fig. 2D). In case of treated wastewater, lower differences were noticed between MEs for the modular SPE protocol and those observed for the HLB sorbent (Fig. 2D); however, the later sorbent was not able to recover short-chain perfluorinated compounds from water samples at neutral pH. Detailed data of MEs for treated wastewater are compiled in Table S3.

The accuracy of the proposed methodology was assessed with recoveries assessed for samples spiked at different concentration levels, and calculated against solvent-based standards. Values obtained for each sample and addition level, including their standard deviations, are given as supplementary information (Table S4). A summary of global recoveries (with minimum and maximum values) observed for raw and treated wastewater samples is compiled in Table 3. Out of 64 compounds considered in this study, 57 and 60 species (in raw and treated wastewater, respectively) showed overall recoveries in the range between 80 and 120%. Therefore, the use of isotopically labeled analogues permitted to compensate problems (EEs below 80%) previously highlighted for those compounds distributed between neutral and basic fractions (IMI and ACE), and most of the species affected by sorption problems. Overall, the global mean of the recoveries for each of the three groups of compounds considered in this study varied between 95 and 100%. In summary, the modular SPE method described in this study provides accuracy values, for the three kinds of compounds, similar to those achieved using previously reported conventional MM methodologies, either focused on the selective extraction of acidic compounds [12, 14], or addressing the concentration and isolation of basic drugs [11], from wastewater samples.

**Table 3:** Average accuracy of the procedure for spiked wastewater samples (*n* = 9 samples spiked at 3 concentration levels: 50, 200, and 1000 ng L^−1^) and estimated procedural LOQs

	Compound	Raw wastewater				Treated wastewater				LOQs
		Average	RSD (%)	Min	Max	Average	RSD (%)	Min	Max	ng L^−1^
Acids	2,4-Dichlorophenoxyacetic acid	94%	5%	89%	100%	102%	1%	101%	104%	20
	4-(2,4-Dichlorophenoxy)butyric acid	93%	6%	86%	97%	96%	5%	91%	100%	20
	Candesartan	77%	5%	73%	82%	88%	8%	80%	95%	5
	Eprosartan	89%	9%	84%	100%	92%	4%	89%	96%	5
	Fenoprop	103%	3%	99%	105%	99%	4%	96%	104%	10
	Irbesartan	106%	6%	100%	111%	105%	6%	99%	111%	2
	Losartan	89%	11%	78%	100%	101%	12%	93%	114%	5
	2-Methyl-4-chlorophenoxyacetic acid	96%	9%	86%	104%	98%	6%	91%	104%	10
	Mecoprop	105%	5%	99%	109%	105%	3%	102%	107%	10
	Olmesartan	90%	4%	86%	94%	101%	11%	92%	113%	5
	Pentafluoropropanoic acid	96%	9%	87%	105%	100%	14%	85%	113%	10
	Perfluorobutanoic acid	84%	4%	80%	87%	104%	13%	93%	119%	10
	Perfluorobutano sulfonic acid	111%	12%	104%	125%	109%	7%	105%	117%	2
	Perfluorooctanoic acid	90%	1%	89%	91%	103%	9%	95%	112%	10
	Perfluorooctano sulfonic acid	96%	4%	91%	99%	100%	4%	97%	104%	5
	Telmisartan	108%	14%	92%	120%	107%	11%	100%	119%	5
	Valsartan acid	100%	13%	87%	112%	101%	18%	81%	116%	5
	Valsartan	98%	10%	89%	109%	96%	9%	86%	103%	2
Bases	Acetamiprid	105%	1%	100%	110%	100%	5%	95%	105%	5
	Amitriptyline	95%	7%	90%	103%	99%	5%	94%	103%	2
	Citalopram	89%	8%	81%	96%	103%	9%	92%	109%	3
	Climbazole	97%	3%	94%	100%	106%	2%	104%	107%	3
	Clomipramine	112%	6%	105%	117%	106%	4%	103%	111%	3
	Cloperastine	117%	3%	113%	119%	110%	4%	106%	115%	5
	Clotrimazole	97%	3%	95%	100%	98%	7%	90%	103%	5
	Fenticonazole	53%	12%	41%	64%	66%	15%	49%	77%	10
	Flecainide	92%	6%	87%	98%	101%	13%	86%	112%	4
	Fluconazole	104%	8%	96%	112%	120%	5%	115%	126%	5
	Haloperidol	86%	7%	80%	93%	95%	5%	89%	98%	5
	Imazalil	102%	3%	98%	104%	98%	8%	89%	105%	10
	Imidacloprid	107%	2%	91%	120%	112%	20%	95%	134%	10
	Lamotrgine	76%	20%	63%	99%	108%	24%	81%	128%	10
	Metconazole	97%	6%	90%	102%	98%	0%	98%	99%	6
	Miconazole	98%	8%	93%	108%	99%	4%	95%	104%	4
	Myclobutanil	95%	1%	95%	96%	102%	4%	97%	105%	2
	N-Desethyl amiodarone	76%	3%	72%	79%	89%	1%	87%	90%	5
	N-Desmethyl citalopram	86%	14%	72%	100%	93%	2%	91%	95%	5
	Norsertraline	102%	9%	97%	112%	104%	7%	99%	112%	10
	O-Desmethyl venlafaxine	86%	20%	73%	109%	90%	10%	79%	97%	3
	Penconazole	95%	3%	92%	98%	101%	9%	91%	108%	2
	Prochloraz	83%	3%	81%	87%	95%	11%	85%	107%	5
	Propiconazole	96%	1%	95%	98%	102%	7%	95%	110%	5
	Propranolol	90%	10%	81%	100%	107%	6%	100%	113%	5
	Sertaconazole	82%	2%	80%	83%	89%	6%	85%	96%	10
	Sertraline	108%	16%	95%	126%	114%	11%	101%	122%	5
	Tebuconazole	94%	8%	89%	103%	102%	6%	96%	106%	5
	Terbutryn	104%	4%	101%	109%	106%	6%	98%	111%	2
	Tetraconazole	96%	3%	93%	100%	101%	3%	99%	105%	4
	Thiabendazole	98%	42%	67%	146%	93%	11%	81%	102%	2
	Tioconazole	129%	9%	123%	140%	108%	5%	102%	112%	10
	Tramadol	101%	7%	93%	105%	107%	9%	97%	112%	2
	Trazodone	85%	8%	76%	92%	87%	12%	78%	100%	10
	Venlafaxine	98%	0%	98%	98%	106%	6%	100%	111%	2
Neutrals	Clothianidin	90%	3%	77%	97%	93%	3%	91%	96%	5
	Cresyl diphenyl phosphate	97%	3%	95%	99%	90%	11%	78%	99%	3
	Dimoxystrobin	109%	2%	108%	110%	102%	5%	99%	108%	1
	Octyl isothiazolinone	106%	5%	101%	112%	96%	15%	79%	108%	5
	Tris(2-chloroethyl) phosphate	79%	1%	78%	79%	78%	18%	63%	99%	10
	Tris(1-chloro-2-propyl) phosphate	105%	1%	98%	111%	100%	9%	90%	108%	10
	Triclosan	94%	0%	93%	95%	94%	1%	92%	94%	5
	Thiamethoxam	98%	0%	95%	102%	96%	4%	94%	101%	3
	Tributoxyethyl phosphate	103%	3%	88%	118%	102%	11%	90%	109%	2
	Tributyl phosphate	86%	1%	74%	93%	103%	12%	95%	116%	10
	Triphenyl phosphate	97%	1%	90%	107%	95%	16%	83%	113%	10

The last column in Table 3 summarizes the LOQs of the method for raw wastewater. For the perfluorinated carboxylic acids and organophosphorus species, the global LOQs were determined by responses observed in procedural blanks. For the rest of compounds, procedural LOQs were controlled by instrumental LOQs (Table 1) and the performance of the SPE extraction step. With the exception of two phenoxy acid herbicides, the procedural LOQs varied between 2 and 10 ng L^−1^. Overall, LOQs shown in Table 3 are in the range of values reported in previous studies using LC–MS/MS as determination technique after SPE either using HLB type [4, 6] or a single MM cartridge [10, 11]. Obviously, none of these previous methods covered the range of polarities considered in the current research.

### Application to wastewater samples

The presence of target compounds was evaluated in six pairs of water samples (inlet and outlet), obtained from four STPs. Positive identifications were based on retention time and Q2/Q1 matches (0.1 min and ± 30%, respectively) with calibration standards. A group of 46 compounds was quantified in at least one of the processed samples. Their concentrations (average values for duplicate extractions) are shown in Table S5. Among them, 33 species reached average levels above, or very close to 20 ng L^−1^, either in raw or in treated wastewater.

**Fig. 3:**
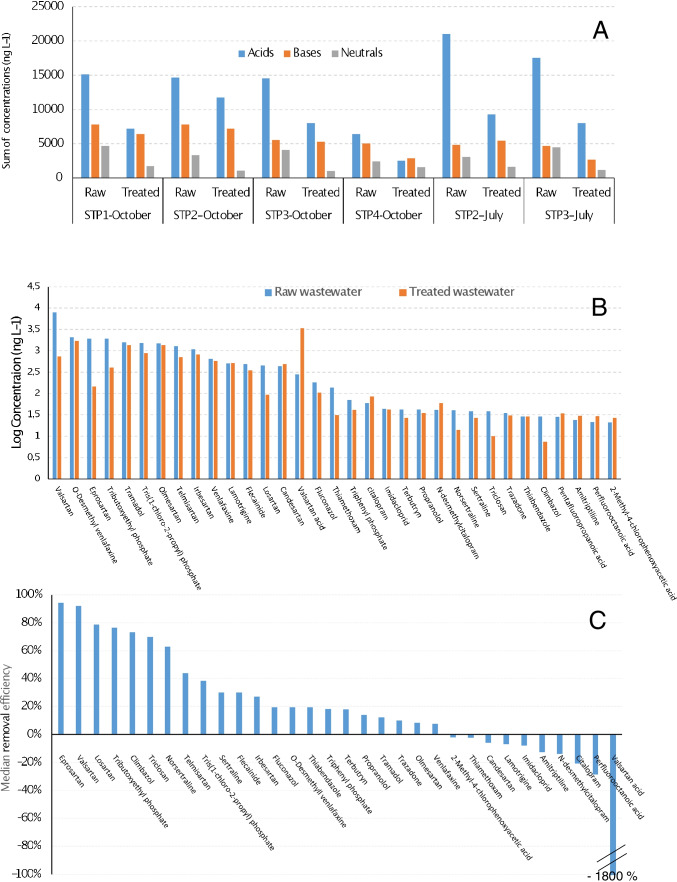
**A** Sum of concentrations (ng L − 1) for acids, bases, and neutrals in raw and treated wastewater from different STPs. **B** Average concentrations for ubiquitous pollutants (logarithmic scale) in raw and treated wastewater. **C** Median removal efficiencies of selected compounds

Figure 3A shows the sum of concentrations for acidic, basic, and neutral pollutants in wastewater samples. The contribution of species detected, but remaining below method LOQs, was not considered. In most of the samples, the sum of concentrations of acids was higher than those of bases and neutrals. On the other hand, the overall concentration of bases was that showing the lower reduction during wastewater treatment. Figure 3B displays the average concentrations (logarithmic scale) of compounds found at levels above 20 ng L^−1^ in raw and treated wastewater. In the first matrix, 16 species showed average levels above 100 ng L^−1^. Within this group, we found two organophosphorus compounds (TCPP and TBEP), several cardiovascular drugs (in most cases ARA-II compounds and flecainide), opioids (such as TRA), different psychiatric drugs, and their human metabolites (VEN, O-DVEN, CIT, and N-DCIT). Among pesticides, terbutryn, imidacloprid, thiamethoxam, and thiabendazole were also ubiquitous in wastewater, with average concentrations in the range from 30 to 100 ng L^−1^

Figure 3C shows the median value of the apparent removal efficiencies for those compounds found above their LOQs in at least four of the six pairs of composite sewage water samples. Compounds are sorted from higher to lower removal efficiencies. Several pollutants displayed very low (below 20%), even negative, removal efficiencies, leading to similar, even higher, concentrations in treated than in raw wastewater. In case of pharmaceuticals, a potential explanation for negative removal rates is de-conjugation during wastewater treatment, as reported for lamotrigine [21]. For moderately lipophilic compounds, negative removal rates might be an artifact caused by differential sorption of these species on particulate matter existing in raw and treated wastewater, either at STPs, or during transport. Whatever the exact source, similar trends have been already reported in case of perfluorooctanoic acid [22]. A particular case of compound generated in the STPs was VALA. This species is a biodegradation product of some ARA-II drugs, particularly of VAL [12]. On average, its concentration in treated wastewater was 10-times higher than in the influent of STPs (Fig. 3B and C). In fact, VALA was the compound showing the highest average concentration in the effluents of the STPs. It is also worth noting that some compounds showing high apparent removal efficiencies (i.e., TBEP, telmisartan, losartan, TCS, and climbazole) have been previously reported in sludge at moderate-to-high concentrations [23, 24]. Thus, sludge sorption might be responsible, at least in part, for the apparent degradation efficiencies depicted in Fig. 3C

The efficiency of the modular SPE protocol to isolate additional compounds in a single fraction, eluted from the combination of SPE sorbents, was assessed using a LC-ESI-QTOF-MS system. To this end, a list of suspected targets was investigated in every SPE fraction from three different raw wastewater extracts. Their normalized responses (Table S6) confirmed trends observed for the previous suite of targets. Compounds with a carboxylic acid, or a stronger acid functionality, were recovered in the fraction from the WAX cartridge, no matter the co-existence of basic moieties in their molecules (e.g., atorvastatin, furosemide, and diclofenac). Slightly (caffeine) and strong basic compounds (cocaine, ephedrine, amisulpride) were trapped by the pure cationic exchange SCX sorbent. Finally, the set of investigated phenols (acetaminophen, benzophenone-3, methyl and propyl paraben) was mostly recovered with neutrals given their weak and null interactions with WAX and SCX sorbents, respectively.

## Conclusions

The modular SPE configuration described in this research permitted the effective concentration of a suite of 64 compounds, with log *D* values comprised between − 1.95 and 4.5 units, from wastewater samples. Compared to an HLB-type sorbent, the described setup allowed the effective retention of relevant groups of polar, anionic pollutants, without requiring acidification of water samples. Considering the obtained MEs, the fraction of bases showed a much lower complexity than that obtained using the HLB-type sorbent. To the best of our knowledge, this study reports for the first time the successful extraction and fractionation of acids, bases, and neutrals from water samples combining commercially available cartridges of different sorbents. In addition to its quantitative applications, the proposed SPE setup might be useful in non-target screening studies, to reduce the number of potential candidates existing in each fraction obtained from the same water sample. Quantitative data obtained for integrated water samples highlighted several pharmaceuticals poorly removed, even generated, during wastewater treatment. These species might serve as markers to assess the impact of urban wastewater in surface water reservoirs.

## Supplementary Information

Below is the link to the electronic supplementary material.

Supplementary file1 (DOCX 625 KB)
