# Supplementary material for: Solid-phase extraction and fractionation of multiclass pollutants from wastewater followed by liquid chromatography tandem-mass spectrometry analysis
Source: Anal Bioanal Chem. 2022 Apr 23;414(14):4149–65. doi: 10.1007/s00216-022-04066-8 (PMC9124662; doi:10.1007/s00216-022-04066-8)
Supplement: Supplementary file 1 — Supplementary file1 (DOCX 625 KB) [file 216_2022_4066_MOESM1_ESM.docx]

Supplementary material to:

**Solid-phase extraction and fractionation of multiclass pollutants from wastewater followed by liquid chromatography tandem mass spectrometry analysis**

V. Fernández-Férnandez, M. Ramil, R. Cela, I. Rodríguez*

Department of Analytical Chemistry, Nutrition and Food Sciences. Research Institute on Chemical and Biological Analysis (IAQBUS). Universidade de Santiago de Compostela, 15782-Santiago de Compostela, Spain.

*Corresponding author

e-mail: [isaac.rodriguez@usc.es](mailto:isaac.rodriguez@usc.es)

Table S1. Summary of LC-ESI-MS/MS determination conditions for the surrogate standards (SSs) employed in the study

|  |  |  | Group |  |  |  | Name | Precursor Ion | Q1 (CE) | Q2 (CE) | Ratio | Ret. Time (min) | Polarity |
| --- | --- | --- | --- | --- | --- | --- | --- | --- | --- | --- | --- | --- | --- |
|  |  |  |  |  |  |  | 2,4-Dichlorophenoxyacetic acid-d_5_ | 224.0 | 164.0 (12) | 127.0 (32) | 0.06 | 6.6 | - |
|  |  |  |  |  |  |  | Irbesartan-d_4_ | 433.3 | 211.1 (24) | 196.2 (24) | 0.19 | 9.7 | + |
|  |  |  | Acids |  |  |  | Perfluorooctanoic acid ^13^C_2_ | 415.0 | 370.0 (17) | 169.0 (5) | 0.15 | 9.9 | - |
|  |  |  |  |  |  |  | Perfluorooctano sulphonic acid ^13^C_8_ | 507.0 | 80.0 (49) | 99.0 (60) | 0.52 | 10.5 | - |
|  |  |  |  |  |  |  | Valsartan acid-d_4_ | 271.1 | 210.1 (20) | 182.1 (36) | 0.5 | 6.6 | + |
|  |  |  |  |  |  |  | Climbazole-d_4_ | 297.1 | 69.1 (24) | 201.1 (16) | 1.05 | 7.3 | + |
|  |  |  |  |  |  |  | Clotrimazole-d_5_ | 282.1 | 170.1 (28) | 247.1 (60) | 0.61 | 8.1 | + |
|  |  |  |  |  |  |  | Flecainide-d_4_ | 419.1 | 305.0 (40) | 398.1 (40) | 0.87 | 6.6 | + |
|  |  |  |  |  |  |  | Imazalil-d_5_ | 302.1 | 69.1 (16) | 158.9 (20) | 1.05 | 7.3 | + |
|  |  |  |  |  |  |  | Imidacloprid-d_4_ | 260.1 | 213.1 (12) | 179.1 (12) | 0.82 | 4.6 | + |
|  |  |  |  |  |  |  | Lamotrogine ^13^C_3_ | 259.0 | 44.0 (32) | 213.9 (40) | 0.31 | 4.8 | + |
|  |  |  | Bases |  |  |  | Miconazole-d_5_ | 422.0 | 160.8 (40) | 162.8 (40) | 0.91 | 9.5 | + |
|  |  |  |  |  |  |  | Myclobutanil-d_4_ | 293.1 | 70.1 (16) | 129.1 (32) | 0.24 | 9.3 | + |
|  |  |  |  |  |  |  | Norsertraline ^13^C_6_ | 281.0 | 158.9 (20) | - | - | 8.4 | + |
|  |  |  |  |  |  |  | Tebuconazole-d_9_ | 317.1 | 70.1 (40) | 124.9 (47) | 0.5 | 10.0 | + |
|  |  |  |  |  |  |  | Tramadol ^13^Cd_3_ | 268.2 | 58.1 (20) | - | - | 4.7 | + |
|  |  |  |  |  |  |  | Venlafaxine-d_6_ | 284.2 | 64.1 (25) | 266.2 (9) | 0.24 | 6.1 | + |
|  |  |  |  |  |  |  | Acetamiprid-d_3_ | 226.1 | 127.0 (27) | 56.1 (12) | 0.38 | 5.7 | + |
|  |  |  |  |  |  |  | Chlothianidin-d_3_ | 253.0 | 172.1 (8) | 131.9 (8) | 0.60 | 5.3 | + |
|  |  |  | Neutrals |  |  |  | Tris(1-chloro-2-propyl) phosphate d_18_ | 345.1 | 103.0 (28) | - | - | 7.8 | + |
|  |  |  |  |  |  |  | Triclosan ^13^C_6_ | 292.8/294.9 | 35.1 (5) | 37.1 (5) | 0.33 | 9.1 | - |
|  |  |  |  |  |  |  | Thiametoxam-d_4_ | 296.0 | 215.1 (8) | 132.0 (24) | 0.37 | 5.0 | + |
|  |  |  |  |  |  |  | Tributyl phosphate-d_27_ | 294.1 | 165.9 (20) | 103.0 (20) | 0.26 | 8.9 | + |

Table S2. Summary of chromatographic conditions employed for the determination of each group of compounds

| Group | Delay column | Analytical Column | Mobile phases | Column  temperature (ºC) | Mobile phase  flowrate (mL min^-1^) | Mobile phase  gradient |
| --- | --- | --- | --- | --- | --- | --- |
| Acids | InfinityLab PFC (4.6 x 30mm), Agilent Technologies | Luna Omega C18 (100mm x 2.1mm, 3µm; Phenomenex) | A: water, 1mM NH_4_F | 35 | 0.350 | 0.00-1.00 min (2%B)  12.00-13.00 min (100%B) 13.10-17.00 min (2%B) |
|  |  |  | B: methanol, 1 mM NH_4_F |  |  |  |
| Bases | None | Zorbax Eclipse Plus C18 rapid resolution column (50 mm x 2.1 mm, 1.8 µm; Agilent Technologies) | A: water, 0.1% FA | 40 | 0.400 | 0.00 min (2%B)  12.00-13.00 min (100%B) 13.10-17.00 min (2%B) |
|  |  |  | B: methanol, 0.1% FA |  |  |  |
| Neutrals | Same as analytical column | Zorbax Eclipse Plus C18 rapid resolution column (50 mm x 2.1 mm, 1.8 µm; Agilent Technologies) | A: water, 0.1% FA | 40 | 0.250 | 0.00-1.00 min (2%B)  9.00-11.00- min (100%B) 11.10-14.00 min (2%B) |
|  |  |  | B: acetonitrile, 0.1% FA |  |  |  |

Table S3. Summary of MEs (%, as normalized responses to those obtained for a solvent-based standard) for spiked extracts of treated wastewater as function of the SPE protocol.

|  |  | Modular SPE | | HLB SPE | |
| --- | --- | --- | --- | --- | --- |
|  | Compound | Mean | RSD | Mean | RSD |
| Acids | 2,4-Dichlorophenoxyacetic acid | 118.9% | 1.2% | 101.2% | 4.0% |
|  | 4-(2,4-Dichlorophenoxy)butyric acid | 114.6% | 2.6% | 90.5% | 2.3% |
|  | Candesartan | 100.8% | 0.4% | 89.1% | 2.4% |
|  | Eprosartan | 110.9% | 1.1% | 117.3% | 8.5% |
|  | Fenoprop | 131.8% | 1.7% | 115.5% | 3.7% |
|  | Irbesartan | 116.4% | 1.3% | 120.1% | 5.7% |
|  | Losartan | 110.7% | 0.3% | 99.6% | 3.4% |
|  | Mecoprop | 124.5% | 2.4% | 105.6% | 4.9% |
|  | 2-Methyl-4-chlorophenoxyacetic acid | 115.9% | 2.3% | 95.2% | 5.1% |
|  | Olmesartan | 104.5% | 1.2% | 88.6% | 2.0% |
|  | Pentafluoropropanoic acid | 106.8% | 1.0% | 108.7% | 2.9% |
|  | Perfluorobutano sulfonic acid | 108.5% | 1.5% | 89.3% | 1.9% |
|  | Perfluorobutanoic acid | 122.4% | 1.3% | 126.5% | 3.9% |
|  | Perfluorooctano sulfonic acid | 111.8% | 1.7% | 113.3% | 7.1% |
|  | Perfluorooctanoic acid | 117.2% | 1.0% | 115.7% | 3.3% |
|  | Telmisartan | 121.2% | 1.5% | 152.0% | 16.7% |
|  | Valsartan | 98.0% | 0.8% | 72.8% | 2.6% |
|  | Valsartan acid | 70.1% | 1.1% | 34.6% | 1.9% |
| Bases | Acetamiprid | 93.4% | 1.2% | 85.4% | 0.2% |
|  | Amitriptyline | 95.1% | 2.9% | 78.3% | 2.0% |
|  | Citalopram | 88.4% | 3.9% | 71.3% | 2.3% |
|  | Climbazole | 93.4% | 3.7% | 77.1% | 1.8% |
|  | Clomipramine | 93.7% | 3.4% | 78.4% | 2.3% |
|  | Cloperastine | 97.5% | 2.9% | 88.3% | 1.6% |
|  | Clotrimazole | 113.3% | 2.6% | 98.8% | 1.5% |
|  | Fenticonazole | 97.7% | 3.8% | 86.1% | 4.3% |
|  | Flecainide | 88.8% | 2.5% | 85.1% | 2.3% |
|  | Fluconazole | 90.0% | 2.7% | 90.5% | 2.5% |
|  | Haloperidol | 90.1% | 2.2% | 79.1% | 2.6% |
|  | Imazalil (Enilconazole) | 95.9% | 4.5% | 79.3% | 1.7% |
|  | Imidacloprid | 66.4% | 7.0% | 81.8% | 0.6% |
|  | Lamotrogine | 70.1% | 3.7% | 44.9% | 3.0% |
|  | Metconazole | 98.6% | 4.1% | 95.7% | 1.0% |
|  | Miconazole | 93.6% | 2.8% | 83.1% | 3.5% |
|  | Myclobutanil | 98.8% | 3.2% | 92.8% | 2.0% |
|  | N-desethylamiodarone | 96.6% | 3.0% | 87.3% | 3.4% |
|  | N-desmethyl citalopram | 78.3% | 4.5% | 61.9% | 2.4% |
|  | Norsertraline | 104.0% | 5.3% | 89.0% | 2.1% |
|  | O-desmethyl venlafaxine | 68.1% | 1.3% | 68.6% | 1.6% |
|  | Penconazole | 99.0% | 4.4% | 88.8% | 1.7% |

Table S3. Cont.

| Bases | Prochloraz | 84.4% | 7.7% | 91.5% | 2.7% |
| --- | --- | --- | --- | --- | --- |
|  | Propiconazole | 99.3% | 3.7% | 97.6% | 1.2% |
|  | propranolol | 81.0% | 3.7% | 75.4% | 1.6% |
|  | Sertaconazole | 93.2% | 3.1% | 80.2% | 2.7% |
|  | Sertraline | 88.5% | 4.3% | 66.9% | 2.7% |
|  | Tebuconazole | 98.8% | 3.4% | 92.5% | 0.6% |
|  | Terbutryn | 96.6% | 2.6% | 82.8% | 2.0% |
|  | Tetraconazole | 99.8% | 3.8% | 97.4% | 1.9% |
|  | Thiabendazole | 60.2% | 1.6% | 61.7% | 1.5% |
|  | Tioconazole | 97.9% | 4.2% | 79.6% | 2.8% |
|  | Tramadol | 92.0% | 2.1% | 85.4% | 1.1% |
|  | Trazodone | 69.8% | 3.0% | 62.5% | 3.2% |
|  | Venlafaxine | 93.1% | 2.6% | 84.7% | 1.3% |
| Neutrals | Clothianidin | 77.1% | 3.9% | 58.2% | 1.4% |
|  | Cresyl Diphenylphosphate | 105.7% | 1.8% | 108.4% | 1.8% |
|  | Dimoxystrobin | 103.2% | 0.4% | 114.1% | 0.3% |
|  | Octyl isothiazolinone | 96.2% | 0.4% | 97.3% | 0.5% |
|  | Tris(2-chloroethyl) phosphate | 77.9% | 4.0% | 62.0% | 2.3% |
|  | Tris(1-chloro-2-propyl) phosphate | 83.3% | 0.6% | 104.2% | 0.6% |
|  | Triclosan | 101.7% | 1.2% | 98.2% | 1.1% |
|  | Thiamethoxam | 121.1% | 5.8% | 91.4% | 1.5% |
|  | Tributoxyethyl phosphate | 96.0% | 0.6% | 106.5% | 1.0% |
|  | Tributyl phosphate | 96.5% | 0.7% | 99.4% | 0.2% |
|  | Triphenyl phosphate | 101.6% | 1.3% | 106.7% | 1.0% |

Table S4. Summary of overall recoveries for wastewater samples spiked at different concentration levels, n=3 replicates per sample and addition level.

| Compound | | 50 ng L^-1^ | | | | 200 ng L^-1^ | | | | 1000 ng L^-1^ | | | |
| --- | --- | --- | --- | --- | --- | --- | --- | --- | --- | --- | --- | --- | --- |
|  |  | Treated wastewater | | Raw wastwater | | Treated wastewater | | Raw wastwater | | Treated wastewater | | Raw wastwater | |
|  |  | Recovery | RSD (%) | Recovery | RSD (%) | Recovery | RSD (%) | Recovery | RSD (%) | Recovery | RSD (%) | Recovery | RSD (%) |
| Acids | 2,4-Dichlorophenoxyacetic acid | 103% | 6% | 93% | 3% | 101% | 4% | 100% | 3% | 104% | 4% | 89% | 3% |
|  | 4-(2,4-Dichlorophenoxy)butyric acid | 98% | 6% | 97% | 9% | 100% | 2% | 97% | 6% | 91% | 3% | 86% | 4% |
|  | Candesartan | 95% | 2% | 82% | 3% | 90% | 2% | 73% | 2% | 80% | 5% | 76% | 3% |
|  | Eprosartan | 96% | 3% | 100% | 2% | 91% | 3% | 84% | 2% | 89% | 4% | 84% | 3% |
|  | Fenoprop | 97% | 8% | 105% | 5% | 96% | 5% | 105% | 3% | 104% | 2% | 99% | 2% |
|  | Irbesartan | 99% | 2% | 111% | 1% | 111% | 2% | 106% | 2% | 104% | 2% | 100% | 1% |
|  | Losartan | 114% | 11% | 100% | 3% | 93% | 3% | 89% | 2% | 94% | 3% | 78% | 3% |
|  | Mecoprop | 107% | 11% | 106% | 9% | 102% | 3% | 109% | 2% | 107% | 3% | 99% | 3% |
|  | 2-methyl-4-chlorophenoxyacetic acid | 98% | 6% | 97% | 6% | 104% | 3% | 104% | 3% | 91% | 5% | 86% | 3% |
|  | Olmesartan | 113% | 4% | 89% | 3% | 92% | 1% | 86% | 2% | 97% | 3% | 94% | 4% |
|  | Pentafluoropropanoic acid | 113% | 13% | 105% | 5% | 101% | 6% | 87% | 5% | 85% | 5% | 96% | 7% |
|  | Perfluorobutano sulfonic acid | 105% | 3% | 104% | 4% | 117% | 3% | 125% | 4% | 105% | 3% | 104% | 3% |
|  | Perfluorobutanoic acid | 101% | 1% | 80% | 6% | 93% | 2% | 87% | 3% | 119% | 5% | 84% | 3% |
|  | Perfluorooctano sulfonic acid | 99% | 7% | 99% | 6% | 97% | 3% | 97% | 3% | 104% | 3% | 91% | 2% |
|  | Perfluorooctanoic acid | 95% | 3% | 89% | 4% | 103% | 6% | 90% | 5% | 112% | 4% | 91% | 2% |
|  | Telmisartan | 101% | 2% | 92% | 2% | 119% | 6% | 120% | 2% | 100% | 1% | 113% | 4% |
|  | Valsartan | 97% | 8% | 89% | 2% | 86% | 2% | 109% | 2% | 103% | 4% | 97% | 7% |
|  | Valsartan acid | 81% | 4% | 112% | 4% | 116% | 2% | 102% | 1% | 106% | 3% | 87% | 1% |
| Bases | Acetamiprid | 105% | 1% | 107% | 4% | 95% | 1% | 110% | 1% | 100% | 3% | 100% | 2% |
|  | Amitriptyline | 99% | 4% | 90% | 3% | 103% | 2% | 92% | 6% | 94% | 3% | 103% | 1% |

Table S4. Cont.

|  | Citalopram | 92% | 2% | 81% | 1% | 107% | 2% | 90% | 4% | 109% | 3% | 96% | 1% |
| --- | --- | --- | --- | --- | --- | --- | --- | --- | --- | --- | --- | --- | --- |
|  | Climbazole | 104% | 4% | 94% | 2% | 107% | 4% | 100% | 6% | 107% | 1% | 97% | 1% |
|  | Clomipramine | 111% | 6% | 114% | 3% | 104% | 1% | 105% | 3% | 103% | 5% | 117% | 3% |
|  | Cloperastine | 115% | 4% | 113% | 5% | 106% | 2% | 118% | 5% | 110% | 6% | 119% | 3% |
|  | Clotrimazole | 90% | 4% | 95% | 2% | 103% | 1% | 100% | 5% | 100% | 1% | 95% | 1% |
|  | Fenticonazole | 73% | 3% | 64% | 11% | 77% | 5% | 41% | 6% | 49% | 8% | 53% | 21% |
|  | Flecainide | 86% | 2% | 87% | 1% | 112% | 2% | 92% | 3% | 104% | 0% | 98% | 1% |
|  | Fluconazole | 119% | 2% | 112% | 1% | 115% | 2% | 105% | 4% | 126% | 7% | 96% | 3% |
|  | Haloperidol | 97% | 11% | 84% | 3% | 98% | 2% | 93% | 6% | 89% | 1% | 80% | 2% |
|  | Imazalil (Enilconazole) | 89% | 10% | 103% | 9% | 100% | 5% | 104% | 8% | 105% | 3% | 98% | 2% |
|  | Imidacloprid | 93% | 15% | 112% | 7% | 134% | 4% | 120% | 9% | 108% | 5% | 117% | 13% |
|  | Lamotrogine | 114% | 8% | 99% | 3% | 81% | 3% | 67% | 4% | 128% | 20% | 63% | 4% |
|  | Metconazole | 98% | 1% | 102% | 3% | 98% | 2% | 100% | 7% | 99% | 2% | 90% | 1% |
|  | Miconazole | 95% | 7% | 94% | 3% | 99% | 4% | 93% | 7% | 104% | 0% | 108% | 3% |
|  | Myclobutanil | 97% | 1% | 95% | 2% | 105% | 2% | 96% | 6% | 104% | 2% | 95% | 2% |
|  | N-desethylamiodarone | 90% | 9% | 79% | 6% | 89% | 4% | 76% | 3% | 87% | 10% | 72% | 11% |
|  | N-desmethyl citalopram | 95% | 4% | 88% | 2% | 91% | 3% | 72% | 3% | 94% | 11% | 100% | 3% |
|  | Norsertraline | 99% | 4% | 97% | 5% | 112% | 4% | 97% | 9% | 101% | 1% | 112% | 5% |
|  | O-desmethylVenlafaxine | 97% | 3% | 109% | 3% | 79% | 1% | 76% | 3% | 94% | 4% | 73% | 3% |
|  | Penconazole | 91% | 3% | 96% | 5% | 108% | 3% | 98% | 6% | 104% | 2% | 92% | 1% |
|  | Prochloraz | 94% | 3% | 81% | 5% | 85% | 6% | 87% | 8% | 107% | 2% | 82% | 2% |
|  | Propiconazole | 95% | 4% | 96% | 2% | 101% | 2% | 98% | 6% | 110% | 5% | 95% | 3% |
|  | propranolol | 113% | 1% | 100% | 3% | 100% | 2% | 81% | 3% | 108% | 2% | 88% | 1% |
|  | Sertaconazole | 85% | 7% | 81% | 5% | 96% | 4% | 80% | 6% | 86% | 4% | 83% | 11% |
|  | Sertraline | 101% | 4% | 95% | 3% | 117% | 6% | 104% | 6% | 122% | 1% | 126% | 8% |

Table S4. Cont.

|  | Tebuconazole | 96% | 3% | 103% | 4% | 106% | 1% | 91% | 6% | 105% | 1% | 89% | 1% |
| --- | --- | --- | --- | --- | --- | --- | --- | --- | --- | --- | --- | --- | --- |
|  | Terbutryn | 111% | 3% | 109% | 3% | 108% | 3% | 101% | 7% | 98% | 3% | 101% | 2% |
|  | Tetraconazole | 99% | 2% | 100% | 4% | 99% | 2% | 96% | 6% | 105% | 8% | 93% | 3% |
|  | Thiabendazole | 102% | 8% | 146% | 2% | 95% | 2% | 81% | 4% | 81% | 13% | 67% | 7% |
|  | Tioconazole | 102% | 4% | 124% | 5% | 109% | 5% | 123% | 8% | 112% | 5% | 140% | 8% |
|  | Tramadol | 97% | 3% | 104% | 3% | 112% | 1% | 93% | 2% | 111% | 2% | 105% | 1% |
|  | Trazodone | 100% | 6% | 92% | 4% | 82% | 4% | 86% | 3% | 78% | 12% | 76% | 3% |
|  | Venlafaxine | 100% | 2% | 98% | 1% | 111% | 2% | 98% | 2% | 107% | 0% | 98% | 1% |
| Neutrals | Clothianidin | 91% | 3% | 77% | 8% | 91% | 2% | 97% | 2% | 96% | 3% | 96% | 3% |
|  | Cresyl Diphenylphosphate | 99% | 6% | 95% | 8% | 93% | 5% | 99% | 13% | 78% | 7% | 96% | 6% |
|  | Dimoxystrobin | 108% | 1% | 110% | 2% | 100% | 3% | 108% | 4% | 99% | 3% | 109% | 5% |
|  | Octyl isothiazolinone | 100% | 8% | 112% | 2% | 79% | 2% | 101% | 4% | 108% | 3% | 106% | 12% |
|  | Tris(2-chloroethyl) phosphate | 99% | 13% | n.e. |  | 73% | 4% | 78% | 6% | 63% | 11% | 79% | 4% |
|  | Tris(1-chloro-2-propyl) phosphate | 108% | 1% | 111% | 1% | 102% | 2% | 104% | 2% | 90% | 4% | 98% | 2% |
|  | Triclosan | 94% | 7% | 93% | 4% | 94% | 3% | 95% | 4% | 92% | 4% | 95% | 4% |
|  | Thiamethoxam | 101% | 3% | 102% | 3% | 94% | 1% | 98% | 3% | 94% | 3% | 95% | 3% |
|  | Tributoxyethyl phosphate | 90% | 2% | 88% | 1% | 108% | 3% | 118% | 5% | 109% | 2% | 103% | 6% |
|  | Tributyl phosphate | 96% | 6% | 92% | 4% | 116% | 2% | 74% | 5% | 95% | 4% | 93% | 3% |
|  | Triphenyl phosphate | 90% | 26% | 90% | 6% | 113% | 4% | 107% | 4% | 83% | 4% | 95% | 3% |

Table S5. Concentrations of compounds in pairs of samples (influent and efluent) obtained from four STPs. Average values for duplicate extractions

| Compound | STP1-Oct. 21 | | STP2-Oct. 21 | | STP3-Oct. 21 | | STP4-Oct. 21 | | STP2- July 21 | | STP3- July 21 | | |
| --- | --- | --- | --- | --- | --- | --- | --- | --- | --- | --- | --- | --- | --- |
|  | R.W. | T.W. | R.W. | T.W. | R.W. | T.W. | R.W. | T.W. | R.W. | T.W. | R.W. | | T.W. |
| Amitriptiline | 22.0 | 65.5 | 51.9 | 44.2 | 22.2 | 9.6 | 19.1 | 7.6 | 22.7 | 46.1 | 6.2 | 7.0 | |
| Citalopram | 40.6 | 152.0 | 83.5 | 100.8 | 57.8 | 63.7 | 56.6 | 37.4 | 53.5 | 100.8 | 65.5 | 46.8 | |
| Climbazol | 46.6 | 12.4 | 12.2 | 4.2 | 15.7 | 3.2 | 71.6 | 16.0 | 5.6 | 3.9 | 18.8 | 4.9 | |
| Flecainide | 695.5 | 521.3 | 766.0 | 621.6 | 463.8 | 309.2 | 352.1 | 154.1 | 332.5 | 350.3 | 334.0 | 159.9 | |
| Fluconazol | 215.8 | 146.8 | 223.1 | 127.7 | 77.5 | 80.3 | 432.0 | 81.9 | 87.3 | 105.1 | 74.9 | 86.0 | |
| Imidacloprid | 46.7 | 50.4 | 27.4 | 48.4 | 59.1 | 56.7 | 9.9 | 21.5 | 66.1 | 40.2 | 48.1 | 35.2 | |
| Lamotrigine | 602.5 | 614.8 | 634.6 | 665.3 | 206.5 | 340.7 | 924.6 | 594.2 | 515.4 | 550.9 | 174.1 | 332.9 | |
| N-desmethyl citalopram | 35.2 | 91.3 | 51.6 | 57.8 | 42.7 | 48.6 | 38.6 | 33.9 | 37.3 | 82.8 | 39.4 | 41.5 | |
| Norsertraline | 48.7 | 22.5 | 63.3 | 10.1 | 30.0 | n.d. | 54.3 | 11.3 | 18.0 | 11.7 | 23.4 | < 10 | |
| O-desmethyl venlafaxine | 2504.0 | 2001.7 | 2832.3 | 2668.4 | 1947.7 | 1969.8 | 1172.7 | 855.6 | 2070.3 | 2344.4 | 1899.7 | 429.7 | |
| Propranolol | 53.3 | 52.5 | 51.4 | 44.2 | 19.7 | 22.4 | 65.1 | 34.8 | 35.3 | 38.6 | 25.3 | 13.8 | |
| Sertraline | 50.8 | 58.4 | 54.0 | 26.6 | 31.3 | 6.3 | 49.0 | 34.3 | 20.2 | 28.5 | 23.3 | 6.2 | |
| Terbutryn | 63.7 | 48.3 | 31.6 | 45.7 | 65.8 | 14.2 | 23.2 | 29.4 | 17.4 | 20.7 | 48.4 | 2.6 | |
| Thiabendazole | 38.2 | 36.6 | 70.1 | 40.1 | 23.3 | 18.8 | 15.4 | 9.3 | 12.9 | 57.5 | 12.7 | 9.7 | |
| Tramadol | 2718.6 | 1970.5 | 2073.6 | 2040.0 | 1477.4 | 1486.0 | 1247.2 | 676.3 | 952.9 | 1130.7 | 985.8 | 809.8 | |
| Trazadone | 47.2 | 41.6 | 44.0 | 15.8 | 48.5 | < 10 | 31.5 | < 10 | 21.9 | 33.2 | 16.3 | < 10 | |
| Venlafaxine | 540.5 | 527.9 | 722.5 | 667.4 | 870.7 | 876.2 | 429.4 | 264.9 | 494.9 | 472.6 | 815.9 | 659.5 | |
| Candesartan | 547.9 | 494.2 | 485.2 | 633.5 | 503.0 | 607.8 | 202.4 | 138.3 | 447.6 | 470.4 | 469.8 | 568.9 | |
| Eprosartan | 2322.1 | 273.5 | 824.5 | 33.7 | 3644.4 | 213.8 | 1493.8 | 16.8 | 888.1 | 227.4 | 2524.3 | 114.0 | |
| Irbesartan | 1521.2 | 905.8 | 1386.2 | 1011.6 | 1260.6 | 1081.5 | 386.8 | 243.0 | 1153.0 | 826.4 | 777.4 | 871.4 | |
| Losartan | 836.0 | 97.6 | 346.1 | 67.7 | 504.6 | 161.8 | 281.5 | 37.5 | 313.3 | 84.1 | 454.2 | 108.8 | |
| 2-methyl-4-chlorophenoxyacetic acid | 10.0 | < 10 | 18.9 | 17.3 | n.d. | < 10 | 13.2 | < 10 | < 10 | 16.3 | 41.1 | 46.3 | |

Table S5. Cont.

| Olmesartan | 1803.6 | 1340.1 | 1734.4 | 1900.7 | 1836.2 | 1779.2 | 843.4 | 524.2 | 1450.3 | 1331.3 | 1312.1 | 1328.4 |
| --- | --- | --- | --- | --- | --- | --- | --- | --- | --- | --- | --- | --- |
| Pentafluoropropanoic acid | < 10 | 24.2 | < 10 | 11.6 | 13.6 | < 10 | 47.9 | 73.7 | < 10 | < 10 | 23.4 | 26.5 |
| Perfluorooctanoic acid | 17.7 | 26.4 | 14.3 | 16.4 | 11.4 | 13.9 | 34.6 | 19.7 | < 10 | n.d. | 29.2 | 69.1 |
| Telmisartan | 2017.3 | 928.7 | 1881.8 | 1053.6 | 1306.5 | 764.9 | 557.7 | 190.2 | 1130.6 | 776.5 | 744.4 | 552.7 |
| Valsartan acid | 516.3 | 2985.2 | 709.6 | 5987.2 | 114.6 | 2748.2 | 63.5 | 1244.9 | 142.4 | 3599.3 | 129.5 | 3722.3 |
| Valsartan | 5544.1 | 169.9 | 7278.4 | 1041.1 | 5392.3 | 666.6 | 2534.8 | 36.8 | 15401.6 | 1928.9 | 10911.8 | 535.2 |
| Tris(1-chloro-2-propyl) phosphate | 1776.6 | 1231.9 | 1628.9 | 483.5 | 1232.8 | 627.3 | 1303.3 | 1260.9 | 892.3 | 741.2 | 2331.0 | 929.6 |
| Triclosan | 33.7 | n.d. | 36.5 | 10.7 | 36.9 | < 5 | 63.8 | 6.7 | 23.4 | 12.4 | 31.7 | n.d. |
| Thiamethoxam | 6.6 | 8.6 | 29.7 | 47.6 | 18.7 | 8.8 | n.d. | 5.6 | 635.0 | 106.5 | 6.9 | 7.7 |
| Tributoxyethyl phosphate | 2818.0 | 457.4 | 1596.8 | 491.4 | 2664.0 | 339.7 | 963.2 | 231.7 | 1542.2 | 732.5 | 2077.7 | 197.1 |
| Triphenyl phosphate | 35.4 | 35.3 | 25.4 | 32.3 | 136.8 | 29.9 | 82.8 | 64.7 | n.d. | n.d. | n.d. | n.d. |

Table S6. Distribution of suspected wastewater pollutants in the fractions obtained from the modular SPE cartridge configuration. Average normalized responses (peak area in each fraction divided by the sum of peak areas in the 3 fractions from each sample) for 3 different raw wastewater samples.

| Compound | Structure | WAX fraction | SCX fraction | Neutrals fraction |
| --- | --- | --- | --- | --- |
| Acetaminophen | 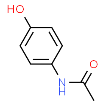 |  |  | 100% |
| Alprazolam | 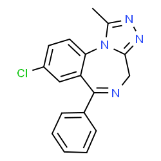 |  | 100% |  |
| Amisulpride | 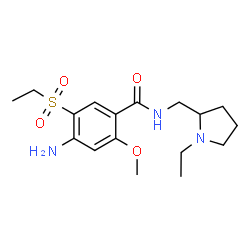 |  | 100% |  |
| Atorvastatin | 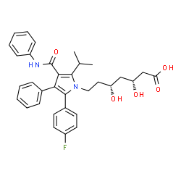 | 100% |  |  |
| Benzophenone 3 | 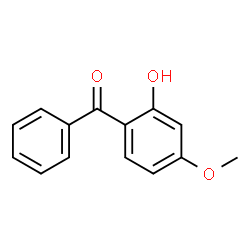 | 7% |  | 93% |
| Benzophenone 4 | 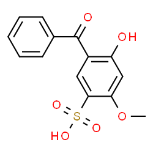 | 100% |  |  |
| Caffeine | 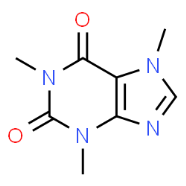 | 1% | 99% |  |
| Carbamazepine | 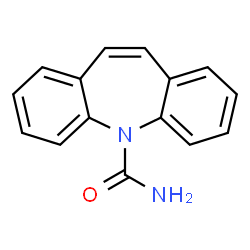 |  | 0% | 100% |
| Cocaine | 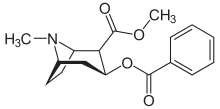 |  | 100% |  |
|  |  |  |  |  |

Table S6. Cont.

| Diclofenac | 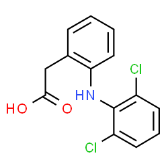 | 100% |  |  |
| --- | --- | --- | --- | --- |
| Ephedrine | 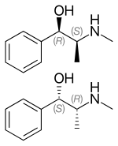 | 1% | 99% |  |
| Furosemide | 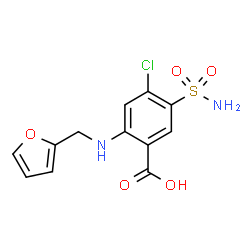 | 100% |  |  |
| Gemfibrozil | 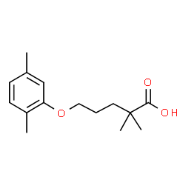 | 100% |  |  |
| Methylparaben | 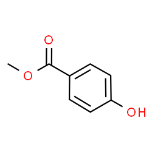 |  |  | 100% |
| Propylparaben | 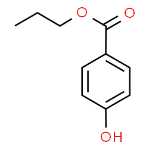 | 23% |  | 77% |
| Trimethoprim | 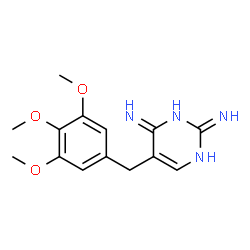 |  | 100% |  |

Fig. S1. Normalized responses for acid compounds as function of the mobile phase modifier, ammonium fluoride (FNH_4_, 1 mM) versus formic acid (FA, 0.1%).

Fig. S2. Separation of analytical peak (1) from mobile phase contamination (2) using delay columns. MRM chromatograms (Q1 and Q2 transitions) for a 0.5 ng mL^-1^ standard of Tris(2-chloro-isopropyl) phosphate (A), Tributoxyethyl phosphate (B), and perfluorooctanoic acid (C).
